# Evaluating the Ability to ID (COVID-19) NOW: a Large Real-World Prospective Evaluation of the Abbott ID NOW COVID-19 Assay

**DOI:** 10.1128/spectrum.00513-22

**Published:** 2022-05-17

**Authors:** K. R. Barker, L. N. Small, D. V. Thai, K. Y. Sohn, L. C. Rosella

**Affiliations:** a Division of Microbiology, Department of Laboratory Medicine & Genetics, Trillium Health Partners, Mississauga, Ontario, Canada; b Department of Laboratory Medicine and Pathobiology, University of Toronto, Toronto, Ontario, Canada; c Institute for Better Health, Trillium Health Partners, Mississauga, Ontario, Canada; d Division of Infectious Diseases, Department of Medicine, Trillium Health Partners, Mississauga, Ontario, Canada; e Department of Medicine, University of Toronto, Toronto, Ontario, Canada; f Division of Pathology, Department of Laboratory Medicine & Genetics, Trillium Health Partners, Mississauga, Ontario, Canada; g Division of Biochemistry, Department of Laboratory Medicine & Genetics, Trillium Health Partners, Mississauga, Ontario, Canada; h Division of Epidemiology, Dalla Lana School of Public Health, University of Toronto, Toronto, Ontario, Canada; Johns Hopkins Hospital

**Keywords:** COVID-19, SARS-CoV-2, rapid testing, ID NOW, POCT

## Abstract

The Abbott ID NOW COVID-19 assay is a rapid point-of-care molecular test for SARS-CoV-2 detection. In theory, it has the potential to decrease turnaround times (TATs) and rapidly facilitate patient flow and triage. Reports for its performance have been mixed, likely due to variations in patient cohorts, preanalytical considerations, and study design. We prospectively evaluated the ID NOW performance against reference reverse transcriptase PCR (RT-PCR) tests, using dual swabs. Patients presented at a large multisite academic hospital with the highest volumes of COVID-19 admissions in Canada. From 1,968 valid swabs, 186 were true positive, 1,760 were true negative, 21 were false negatives, and 1 was false positive. At 10.5% positivity rate, the positive and negative predictive values were 99.5% and 98.8%, respectively. This led to a modest increase in the pretest probability in this cohort of individuals presenting <7 days of symptom onset. The mean times from collection to laboratory receipt and receipt to reporting were 31 and 23 min, respectively. This reduced TAT observed in our study may assist with triage of admitted patients and breaking the chain of transmission through immediate notification of status. We also observed how test performance changed with prevalence, and thus, how the test is used to “rule in” or “rule out” disease must be considered. Although the ID NOW is regarded as a rapid test, it is not high throughput and requires rapid transportation times (<1 h) that may not be plausible in large centers. The utility of this test should be considered with the observed TAT and interpreted in the context of limitations discussed.

**IMPORTANCE** Rapid testing for COVID-19 has been recognized as one potentially important measure in managing the pandemic. However, these rapid tests vary grossly in their performance and their applicability. There have been many studies evaluating the performance of rapid tests for SARS-CoV-2 detection. However, they are frequently not prospective, and patients are not simultaneously swabbed to compare the reference standard RT-PCR. Previous ID NOW study findings are mixed, which may be due to various factors, including patient, epidemiological, and preanalytical considerations. It is critical to consider how the pretest and posttest probabilities and epidemiological factors may affect the performance as the community prevalence of disease fluctuates during this highly dynamic pandemic. We consider how the ID NOW may be utilized in different settings, with considerations of public health and infection control and prevention risk tolerance.

## INTRODUCTION

Approximately 400 million cases and 5.8 million deaths worldwide have been attributed to the coronavirus disease 2019 (COVID-19) pandemic (1). Within Canada, there have been approximately 3.1 million cases and 35,000 deaths (2, 3). The gold-standard detection of SARS-CoV-2 and diagnosis of COVID-19 is through the collection of a nasopharyngeal swab (NPS) and further detection of SARS-CoV-2 RNA by use of reverse transcriptase PCR (RT-PCR). However, these tests require laboratory expertise, may be cumbersome, and, in some cases, may lead to longer than ideal turnaround times (TATs), causing disruptions with the patient flow at the hospital and potentially increasing risk of secondary transmission.

Rapid diagnosis of COVID-19 through the use of point-of-care tests (POCTs), in theory, has the capability of reducing TATs and more rapidly facilitating patient movement to appropriate care locations (e.g., COVID-19 units) and triage. Rapid molecular testing may add considerable benefit in situations where patients with respiratory symptoms compatible with COVID-19 require a rapid decision made regarding their care and infection control triage. It may also benefit outbreak settings where a known exposure has occurred. Delays in either of these scenarios pose major challenges for patient care and secondary transmission.

The Abbott ID NOW COVID-19 (Abbott Diagnostics Inc., USA) is a rapid (<15 min) molecular test with a small footprint and is Health Canada approved for the diagnosis of COVID-19 in symptomatic individuals within 7 days of symptom onset (4, 5). The assay is an isothermal nucleic acid amplification technology that detects the RNA-dependent RNA polymerase (RdRp) gene of SARS-CoV-2 and a propriety internal control. The assay requires the collection of a nasal, nasopharyngeal, or throat swab and further testing of the dry swab without elution in viral transport media. Swabs should be tested within 1 h of collection.

To date, reports of diagnostic sensitivity and specificity have been mixed, likely due to large variability in study design and patient cohorts (symptomatic versus asymptomatic, high versus low pretest probability, etc.) (6–15). A recent Cochrane review included five studies for the ID NOW COVID-19 test (12). Summary diagnostic sensitivity was 76.8% (95% confidence interval [CI], 72.9% to 80.3%), while diagnostic specificity was 99.6% (95% CI, 98.4% to 99.9%). Other factors that need to be considered for variation in test parameters include, but are not limited to: (i) specimen type; (ii) pretest probability; (iii) where the patient is in the course of infection; (iv) viral load; (v) severity of disease; (vi) transportation time and conditions; (vii) specimen quality; (viii) community prevalence/positivity rate; (ix) vaccination status; and (x) comorbidities.

Our center, a 1,400-bed multisite community/academic hospital with some of the highest volumes of COVID-19 admissions in Canada, looked to implement rapid molecular detection (using the ID NOW COVID-19 test) of SARS-CoV-2 in the emergency department (ED) for symptomatic individuals within 7 days of symptom onset to assist in rapid triage of positive patients. The ID NOW COVID-19 test was previously validated in the laboratory, and so, this prospective study was deemed to determine the test performance and utility against reference standard RT-PCR from NPSs. In addition, we looked to consider how pre- and posttest probability, as well as epidemiological factors, may affect performance as the community prevalence of disease fluctuates during the pandemic.

## RESULTS

### Overall performance.

Between 10 May and 25 June 2021, a total of 2,244 dual swabs were taken with the instructions to only collect from symptomatic individuals presenting at either ED with symptoms compatible with COVID-19 and onset within 7 days. Ten deep nasal swab specimens resulted as invalid on the ID NOW COVID-19 assay, resulting in 2,234 total dual swabs giving valid results on both assays. Several patients did not meet the inclusion criteria yet had swabs taken for ID NOW: 81 swabs were from asymptomatic individuals; and 195 swabs were from individuals presenting >7 days since symptom onset. This resulted in a total of 1,968 swabs included in the analysis. Overall, there were 186 true positives (TPs), 1,760 true negatives (TNs), 21 false negatives (FNs), and 1 false positive (FP). This resulted in a diagnostic sensitivity of 89.9% (95% CI, 85.0% to 93.3%) and diagnostic specificity of 99.9% (95% CI, 99.7% to 100%). The percent positivity of the cohort at the time of the study was determined to be 11.0%, resulting in a positive predictive value (PPV) of 99.5% (95% CI, 97.0% to 100%) and negative predictive value (NPV) of 98.8% (95% CI, 98.2% to 99.2%). The likelihood ratio was determined to be 1,582. Results were not found to be assigned by chance alone (*P* < 0.0001, Fisher’s exact test). The paired discordant difference between the two methods was also found to be statistically significant (chi-square = 16.41; *P* < 0.0001; McNemar’s test).

**FIG 1:**
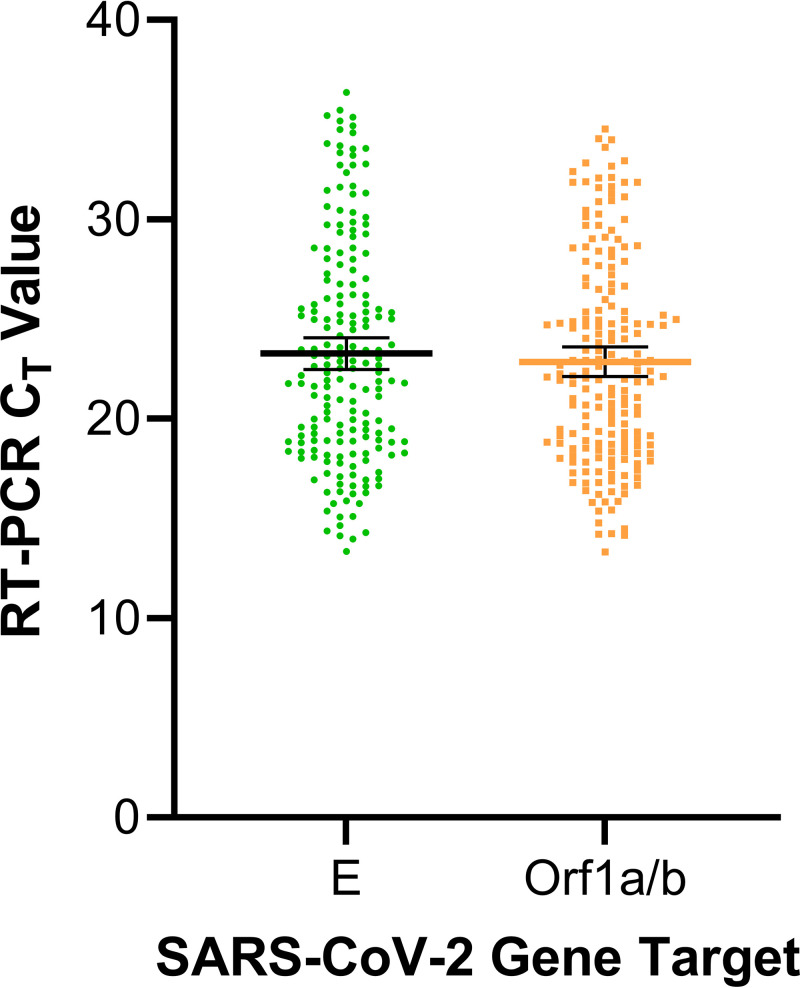
SARS-CoV-2 true-positive specimens by the Abbott ID NOW COVID-19 test and their representative crossing threshold (*C_T_*) values determined by the Roche cobas 6800 cobas SARS-CoV-2 RT-PCR assay. Error bars represent the mean *C_T_* value and the 95% confidence interval. *C_T_*, crossing threshold; E, envelope gene; Orf1a/b, open reading frame 1a/b; RT-PCR, reverse transcriptase-PCR.

Although the reference RT-PCR is a qualitative test, the crossing threshold (*C_T_*) value for each target can be considered to be used as a proxy for viral load but cannot be used to determine viral load without standardization. The mean *C_T_* value for the TPs was determined to be 23.3 (95% CI, 22.5 to 24.1) for the E gene and 22.9 (95% CI, 22.1 to 23.6) for the Orf1a/b gene; see Fig. 1.

### Investigation of false positives.

There was a single patient specimen that was found to be FP by the ID NOW COVID-19 test. The paired NPS was repeated from the primary specimen held at 2 to 8°C within 24 h on the GeneXpert Xpert Xpress SARS-CoV-2/Flu/RSV assay, as well as repeated on the cobas SARS-CoV-2 assay. Both resulted in negative results. Therefore, we sought to determine the likelihood or posttest probability of the patient having acute COVID-19. The patient, a 23-year-old female, presented in the ED at day 7 with mild headache, fever, and cough, with no reported shortness of breath (SOB), ageusia, anosmia, chest pain, myalgia, or malaise. The patient had unremarkable findings on chest X ray, is unvaccinated for COVID-19, and had a history of a negative SARS-CoV-2 RT-PCR test 4 days prior. The patient was subsequently discharged without COVID-19-specific treatment, with a diagnosis of COVID-19.

### Investigation of false negatives.

**FIG 2:**
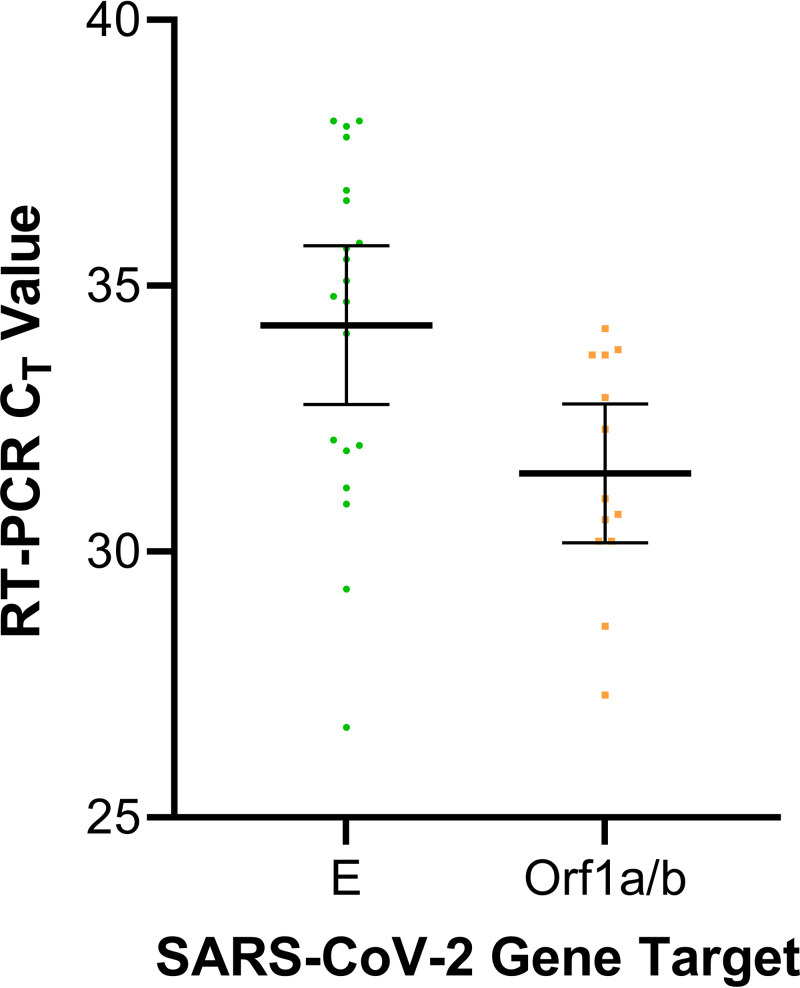
SARS-CoV-2 false-negative specimens by the Abbott ID NOW COVID-19 test and their representative crossing threshold (*C_T_*) values determined by the Roche cobas 6800 cobas SARS-CoV-2 RT-PCR assay. Error bars represent the mean *C_T_* value and the 95% confidence interval. *C_T_*, crossing threshold; E, envelope gene; Orf1a/b, open reading frame 1a/b; RT-PCR, reverse transcriptase-PCR.

There were a total of 21 FNs by the ID NOW COVID-19 test. The mean *C_T_* value determined by the cobas SARS-CoV-2 assay was 34.3 (95% CI, 32.8 to 35.8) for the E gene and 31.5 (95% CI, 30.2 to 32.8) for the Orf1a/b gene; see Fig. 2.

We therefore sought to determine the likelihood or posttest probability of the patient having acute COVID-19 by reviewing the patient charts for the discrepant results; see Table 1. From the 21 FNs, 2 patients had no symptoms of COVID-19 (cellulitis, gastroenteritis) but were listed as symptomatic at the time of ordering, so they are included in the analysis. As noted, the mean *C_T_* value of the FNs was 34.3 (95% CI, 32.8 to 35.8) for the E gene. A total of 7/21 FN specimens produced a *C_T_* value of <33, ranging from 26.7 to 32.1. Only one of these patients tested positive for SARS-CoV-2 previously, with the other six without a history of previous COVID-19. Four patients were admitted to the hospital, while the other three were discharged. Of those four patients, one had a history of COVID-19, one has unknown history, and two had a documented known exposure event(s).

**TABLE 1:** ID NOW false-negative patient specimens compared to reference RT-PCR^*a*^

Characteristic^*b*^	No. of patients	% of patients
Admitted patients (mean no. of days)	10 (15)	47.6
Exposure Hx		
Confirmed	8	38.1
Previously positive	4	19.0
Inconclusive	2	9.5
None	7	33.3
Symptom		
Fever	13	61.9
Cough	10	47.6
SOB	12	57.1
Ageusia	3	14.3
Anosmia	2	9.5
CXR findings	11	52.4
Rx	2	9.5
Vx status		
Vaccinated	4	19.0
Unknown	7	33.3
Unvaccinated	10	47.6
Px Dx COVID-19	9	42.9
Final Dx COVID-19	20	95.2

a CXR, chest X-ray; Dx, diagnosis; Hx, history; Px, presenting; Rx, COVID-19 treatment; SOB, shortness of breath; Sx, symptom; Vx, COVID-19 vaccination.

b Patient admission details, exposure details, symptomology, diagnosis at the time of presentation, vaccination status, treatment history, and final impressions are shown.

## DISCUSSION

After successful verification and validation, our center recently implemented rapid molecular detection (using the ID NOW COVID-19 test) of SARS-CoV-2 in the emergence department (ED) for symptomatic individuals within 7 days of symptom onset to assist in rapid triage of positive patients. We investigated the prospective post-live test performance and utility against reference standard RT-PCR from NPSs and how the pretest probability, posttest probability, and epidemiological factors may affect the performance as the prevalence of disease fluctuates during the pandemic.

The overall performance of the ID NOW COVID-19 test was strong for the Health Canada-approved case use of symptomatic individuals with COVID-19 compatible symptom onset within 7 days (high pretest probability for COVID-19). We hypothesize that we may have shown a higher diagnostic sensitivity (referred to as positive percent agreement in some studies) versus many studies in the literature because those studies recruited other case uses/patient cohorts (e.g., asymptomatic, symptoms >7 days of onset, etc.) (8, 9, 11, 12). Our study does, however, reflect similar results to performance characteristics of others (7, 12, 13, 16). Diagnostic specificity (referred to as negative percent agreement in some studies) is reflective of these studies. The vast majority of false-negative (FN) results (*n* = 21) had high *C_T_* values (proxy for low viral load), with a mean *C_T_* of 34.3 (95% CI, 32.8 to 35.8) for the E gene. Of the 21 FNs, at least 2 should not have been tested, as they were outside the patient case use (symptom incompatible with COVID-19). Seven of the 21 FNs were below of the 95% confidence interval of *C_T_* value of 33.0. *C_T_* values can only be regarded as a proxy for viral load without being standardized and can vary due to multiple factors (including type of swab used, transport media, method and quality of specimen collection, PCR assay, how the PCR threshold line is determined, host response, etc.) (17–20). However, several attempts have been made to determine correlating *C_T_* values with infectivity, giving variable results (21–27). We therefore feel that any extrapolation as to whether these 21 patients are infectious or not cannot be verified by the information we have collected this study. The swab type (deep nasal) cannot be ruled out as a contributor to the reduced diagnostic sensitivity of the ID NOW versus RT-PCR (NPS). Studies have shown similar diagnostic sensitivities but, in some reviews, have not been statistically significant (28–30). This study, however, reflects a true clinical scenario, whereby a smaller/remote center is likely to use the swab provided in the kit (throat, nasal) and recommended in the product’s instructions for use (IFU), rather than provide and undertake an NPS collection. In addition, as found during our clinical validation study, compliance was too low for NPS versus deep nasal. We therefore accepted a slight reduction in diagnostic sensitivity for increased compliance. Lastly, as shown in other studies (including our verification/analytical study [data not shown]), the assay itself has a lower analytical sensitivity, which excludes the contribution of swab type (11, 13, 31–34).

Although the diagnostic sensitivity was 89.9%, the NPV was 98.8% in this study. The positivity rate for this study population was 10.5%, leading to a pretest probability of not being COVID-19 of 89.5%. The posttest probability of a negative result is calculated as 98.8%, based on a negative likelihood ratio of 0.102. This corresponds to a moderate increase on the pretest probability of a negative result, illustrating some use to rule out disease in this patient case use. Multiple factors need to be considered that will affect the test performance and usefulness in this setting. In our study and rollout, we utilized the following: (i) patient cohorts were strictly enforced to the best of our ability, but in some centers, there may be deviation from the case use, as seen in 266 of our specimens; (ii) the specimen collected was a deep nasal swab, which has adequate but lower sensitivity than an NPS, but alternative specimen types may reduce sensitivity; (iii) swabbing was performed by highly skilled nurse practitioners, whereas in some circumstances, alternative, less highly trained people may be utilized; (iv) specimens were transported within 1 h to the laboratory for rapid testing as per the manufacturer’s recommendation, whereas realistically, this may not be possible in a busy ED; and (v) the ID NOW COVID-19 test was being performed by skilled medical lab professionals under strict aseptic conditions, whereas in some cases, the test is performed in the ED by nonmedical lab professionals and in “dirty” areas prone to cross-contamination.

Diagnostic specificity of the ID NOW COVID-19 test in this patient cohort was found to be excellent at 99.9% (95% CI, 99.7% to 100%), with a PPV of 99.5% (95% CI, 97.0% to 100%). The mean TAT from collection to receiving into the laboratory was 31 min, and from receiving to reporting was 23 min. For the same cohort during this period but for RT-PCR, the mean TAT from collection to receiving into the laboratory was 2 h 49 min, and from receiving to reporting was 5 h 7 min. TATs for reference RT-PCR, however, can vary greatly from 6 h to >48 h, depending on transportation time, capacity, and information management. This rapid TAT should greatly assist with the triage of SARS-CoV-2-positive patients who are admitted, as well as informing discharged patients of their positivity status prior to leaving the ED, thereby potentially breaking the chain of transmission more quickly through the immediate notice of a positive COVID-19 status.

The positivity rate or incidence rate of the population may influence the test performance. During this study period, the region where the study was performed was in the early stages of the third wave. At the beginning of this study, Peel’s prevalence was 241.2 cases per 100,000, just shy of its peak the week prior at 313.7 cases per 100,000. By the end of the study period on 25 June 2021, the prevalence had dropped to 3.6 cases per 100,000. Considering the community prevalence is an important factor to consider, however, for the utilization of this test in the ED for symptomatic individuals consistent with COVID-19, one should consider the prevalence or positivity rate in this precise case use cohort. We consider two simple scenarios where the positivity rate doubles and the positivity rate quarters; see Table 2. As the positivity rate or pretest probability increases, the NPV decreases but may still be acceptable, depending on risk tolerance. As the positivity rate or pretest probability decreases, the NPV increases to excellent levels, but the PPV starts to decrease. The medical team needs to strongly consider how this might affect the reliability of the test and whether it can be used to rule in or rule out disease. These considerations need to be individually balanced with lab capacity and TAT for reference RT-PCR. There are many considerations that are leftover that may impact test NPV and PPV, including: (i) the effect of public health interventions; (ii) the effect of vaccination rollout and potential waning immunity; and (iii) the effect of other circulating viruses that present with similar symptomology (e.g., influenza A).

**TABLE 2:** Mock examples of how the change in prevalence/pretest probability affects the positive and negative predictive values of the Abbott ID NOW COVID-19 test in symptomatic individuals consistent with COVID-19 within 7 days of symptom onset^*a*^

Reference RT-PCR	ID NOW COVID-19		Sensitivity (%)	Specificity (%)	PPV (%)	NPV (%)
Positivity rate, 10.5%^*b*^	No. positive	No. negative				
No. positive	186	1				
No. negative	21	1,760				
			89.9	99.9	99.5	98.8
Positivity rate, 21.0%^*b*^						
Positive	372	1				
Negative	42	1,553				
			89.9	99.9	99.7	97.4
Positivity rate, 2.7%^*b*^						
Positive	48	2				
Negative	6	1,912				
			88.9^*c*^	99.9	96.0	99.7

a NPV, negative predictive value; PPV, positive predictive value; RT-PCR, reverse transcriptase-PCR.

b Positivity rate determined by percent positive by reference RT-PCR of total tested (1,968).

c Sensitivity adjusted very slightly to accommodate the whole number of patients/swabs.

How the ID NOW may be utilized in different settings (including applications not approved by health regulators) and whether the performance characteristics are acceptable may depend on risk tolerance. Recently, COVID-19 diagnostics are being considered for practices such as “stay to work” or “return to work” after a specific isolation/quarantine period has been observed from an epidemiological or microbiological diagnosis of COVID-19 (35). The limit of detection of the ID NOW has been shown in multiple studies to be in a “gray zone” for infectivity (a *C_T_* value of around 30 on our in-house RT-PCR assay) (11, 13, 31–34). Although a positive result is likely to indicate a true positive, a negative result must be taken with great caution. One must consider the risk tolerance case by case. Considerations may include: (i) the potential reduction in risk to tolerable levels by implementing stricter public health and infection control practices (i.e., N95 masks, work isolation, etc.); (ii) whether the individual works with vulnerable individuals; (iii) whether the individual works in a higher-risk environment; (iv) the variation in host factors between individuals and how they may affect the viral dynamics; and (v) the vaccination status of the individual.

In addition to evaluation of performance, the ID NOW was implemented in an effort to improve movement and treatment of suspected COVID-19 patients. The high PPV of the rapid test lends itself well to quick confirmation of diagnosis and initiation of COVID-19-specific therapies. Furthermore, expeditious transfer of confirmed patients out of general care areas and into COVID-19-specific care areas was facilitated using this system. Prior to implementing the rapid tests, suspected COVID-19 patients would often remain in the ED for prolonged periods awaiting confirmation with RT-PCR, which potentially delayed care and increased chances of exposing other patients. At times during the height of the pandemic, our center saw over 20 new COVID-19 patients in our ED in a day. Rapidly confirming their diagnosis had a positive impact on their care and the safety of staff and other patients and helped prevent patent movement gridlock.

During the test evaluation period, although a positive ID NOW test did trigger COVID-19 clinical management protocols for a patient, a negative ID NOW test was not used as an action trigger. Only a negative RT-PCR test would change any infection control status of a patient. Although the NPV of the test was very good, some FNs were detected. As such, decisions around infection control protocols based on the single negative rapid result should be made with caution, especially in the setting of symptoms compatible for COVID-19.

In this prospective study with randomized control trial (RCT) confirmation, we show that the ID NOW COVID-19 test is an excellent rapid molecular test to rule in disease in symptomatic individuals presenting at the ED with symptoms compatible with COVID-19 with onset within 7 days. It may also be considered to rule out disease but should be done with careful considerations of clinical and epidemiological factors, with the pretest and posttest probabilities taken into strict consideration. Although the ID NOW COVID-19 test is regarded as a rapid test (less than 15 min), it is not high throughput (one test per machine at one time) and requires rapid transportation times (within 1 h) that may not be plausible in large centers in normal settings. In addition, the “dry” swab may not be able to be used after testing, and so, downstream processing, such as whole genome sequencing, may not be possible unless an appropriate validation for the residual dry swab is done or a separate swab is collected (16). Any consideration for its application to alternative patient case uses such as outbreak settings or asymptomatic screening should be addressed with appropriate validation. Consideration needs to be undertaken in special patient settings such as remote areas without an accredited laboratory and long transportation times to reference laboratories. Although test parameters are not equal to reference RT-PCR, one may need to balance the rapid TAT and interpret the results in the context of these known limitations. Where microbiologist expertise is not easily accessible in these populations, we encourage the support of local or provincial networks with quality assurance and interpretation.

In summary, this is one of the largest studies that we know of that included >2,000 patients with >200 positives and obtained 2 simultaneous swabs for dual testing on the ID NOW COVID-19 test and reference RT-PCR. Unique to our study is the further investigation of FP and FN results within our study to determine the likelihood of them being clinically significant, as well as simultaneously hypothesizing the roll of the ID NOW COVID-19 test at various periods on the epidemiological curve of the pandemic.

## MATERIALS AND METHODS

### Specimen collection.

One deep nasal swab and one NPS were collected simultaneously from individuals who presented at the ED with symptomology and history compatible with COVID-19 within 7 days of symptom onset. Individuals were recruited between 10 May and 25 June 2021 from two large EDs at Mississauga Hospital and Credit Valley Hospital as part of Trillium Health Partners (THP), Mississauga, Ontario, Canada. Deep nasal swabs were placed in sterile containers without transport media, while NPSs were placed in cobas PCR media. The deep nasal swabs were transported at room temperature immediately to the biochemistry laboratory to be tested within 1 h, while the NPSs were transported at room temperature to the microbiology laboratory and stored at 2 to 8°C for up to 24 h prior to testing. NPS specimens were stored at 2 to 8°C until resulting and, if positive by reference RT-PCR, were stored at −80°C if held >24 h postreporting.

### Study design.

Individuals were prospectively included during the period that the ID NOW COVID-19 test was live in the ED at THP. Only individuals presenting with COVID-19-compatible symptoms within 7 days of initial symptom onset were included in the study. Symptomology and symptom onset data were collected at the time of collection and entered into the requisition as per routine clinical care. During the analysis phase, these details were extracted from the laboratory information system, and those not meeting the criteria (i.e., “asymptomatic”) were not included in the analysis. NPS specimens were tested by reference RT-PCR, which was, in this case, the Roche cobas 6800 cobas SARS-CoV-2 RT-PCR assay. Deep nasal specimens were tested by the Abbott ID NOW COVID-19 test within the manufacturer-recommended 1 h since collection. FP results were tested by an alternative reference RT-PCR, Cepheid GeneXpert Xpert Xpress SARS-CoV-2/Flu/RSV assay within 24 h.

### Roche cobas 6800 cobas SARS-CoV-2.

The Roche cobas 6800 cobas SARS-CoV-2 assay is a Health Canada-approved, fully automated, high-throughput “closed-system” real-time RT-PCR assay for the detection of SARS-CoV-2 from nasal swabs, NPSs, and oropharyngeal swabs. The assay detects two regions of the SARS-CoV-2 genome, open reading frames 1a and 1b (Orf1a/b) gene, and the envelope (E) gene. A selective RNA internal control is included as a noncompetitive sequence with no homology with coronaviruses. An exogenous spike external positive control [poly(rA) noninfectious plasmid DNA containing SARS-CoV-2 sequence and noninfectious plasmid DNA containing pan-*Sarbecovirus* sequence] and negative control [poly(rA) RNA] were included on each run. Validation of results was performed automatically by the cobas 6800 software based on negative- and positive-control performance. The assay was performed as per the manufacturer’s IFU. In brief, primary specimen tubes were placed directly on the cobas 6800, with a minimum input volume of 1 mL, and extraction and amplification were undertaken automatically.

### Cepheid GeneXpert Xpert Xpress SARS-CoV-2/Flu/RSV.

The Cepheid Xper Xpress SARS-CoV-2/Flu/RSV is a Health Canada-approved, multiplex real-time RT-PCR assay that provides an on-demand, “random-access” molecular near-point-of-care detection method for SARS-CoV-2, influenza A, influenza B, and respiratory syncytial virus (RSV) for the detection of viral nucleic acids in NPSs, nasal swabs, and nasal washes/aspirate specimens collected from individuals suspected of respiratory viral infections. The assay detects the nucleocapsid (N2) gene and E gene from SARS-CoV-2 and provides a single combined crossing threshold (*C_T_*) value. A sample processing control (SPC) and probe check control (PCC) are included as internal controls. ZeptoMetrix NATtrol Flu/RSV/SARS-CoV-2 positive control (NATFRC-6C; ZeptoMetrix, USA) and negative control (NATCV9-6C) were used as external controls for each shipment and lot of Xpert Xpress cartridges. Validation of results was based on negative- and positive-control performance. The assay was performed as per the manufacturer’s IFU. In brief, 300 μL of NPS specimen was transferred into the cartridge, closed, placed into the GeneXpert module, and the assay started.

### Abbott ID NOW COVID-19.

The Abbott ID NOW COVID-19 is a Health Canada-approved rapid molecular isothermal nucleic acid amplification technology for the detection of SARS-CoV-2 from NPSs or nasal or throat swabs from symptomatic individuals compatible with COVID-19 within 7 days of symptom onset. The assay detects the RdRp segment of the SARS-CoV-2 RNA by use of fluorescently labeled molecular beacons. It is a qualitative test that provides a positive, negative, or invalid test result for SARS-CoV-2. An internal control is included to detect sample inhibition and assay reagent function.

As supplied by the manufacturer and recommended by the IFU, a positive-control swab containing inactivated influenza A and B viruses (AWLB190415; Abbott Diagnostics, USA) and a sterile foam tip applicator negative control (model no. 25-1506; Puritan Medical, USA) were used as external controls for each shipment and lot of ID NOW reagents/materials (test base, sample receive and transfer cartridge). Air-dried swabs of inactivated SARS-CoV-2 virus culture (Sunnybrook Health Sciences Centre, Canada) in Microbix DxTM viral transport medium (catalog no. VTM-640-03; Microbix Biosystems, Canada) with a final concentration of 1 × 10^1^ 50% tissue culture infective dose (TCID_50_)/mL and REDx FLOQ SARS-CoV-2 swab negative control (RED-S-99-M4; Microbix Biosystems, Canada) were used as daily external control to detect assay reagent function, sample interference, and sample cross-reactivity (36). The assay was performed as per the manufacturer’s IFU. In brief, the test base (which contains lyophilized reagents for targeted amplification and the internal control) and the sample receiver (which contains 2.5 mL of elution buffer) are inserted into the ID NOW and warmed up by the instrument. The patient’s swab is mixed in the sample receiver for 10 s. The transfer cartridge collects sample from the sample receiver, and then it is dispensed into the test base. The ID NOW lid is closed, and the assay is started.

### Statistical analysis.

Statistical analysis was performed using Prism v.9.2.0 (GraphPad Software LLC, CA, USA). Overall performance was determined using a contingency table to facilitate sensitivity, specificity, positive predictive value, and negative predictive value calculations. Two-sided Fisher’s exact test was performed to determine statistical significance (α = 0.05), and confidence intervals were calculated using Wilson-Brown.

### Institutional review board/IACUC approval.

Trillium Health Partners Research Ethics Board (REB) determined this project as not being classified as human subjects research, and it therefore was determined as not requiring REB review.
